# Experiencing the Untouchable: A Method for Scientific Exploration and Haptic Fruition of Artworks Microsurface Based on Optical Scanning Profilometry

**DOI:** 10.3390/s21134311

**Published:** 2021-06-24

**Authors:** Sara Mazzocato, Claudia Daffara

**Affiliations:** Department of Computer Science, University of Verona, Strada le Grazie 15, 37134 Verona, Italy; sara.mazzocato@univr.it

**Keywords:** optical microprofilometry, conoscopic holography, 3D printing, tactile fruition, artwork diagnostics

## Abstract

The experience of an object derives not only from the sight but also from the touch: a tactile exploration can reveal the smallest information trapped within the surface up to our tactile detective threshold. Starting from the importance of this observation in the case of works of art, this research demonstrates the use of conoscopic holography sensors for high-quality acquisition of the surface of artworks (up to the micro-scale) suitable also to 3D printing. The purpose is twofold, allowing for the tactile use of the artwork, which is otherwise impossible, for visually impaired people and for new use in regard to scientific information purposes. In detail, the workflow to obtain a 3D printed replica of multiscale and polychrome artworks suitable for the haptic fruition is validated, but the potential of the tool as an innovative resource for scientific visualization of the microsurface of the artwork for conservation issues is also demonstrated. The validation was performed on notable Italian masterpieces, such as Donatello’s “Death Cristh” bronze relief in Padua and the Tintoretto painting “St. Martial in Glory with the Saints Peter and Paul” in Venice.

## 1. Introduction and Background

Our museums and churches have plenty of wonderful works of art exposed to the people that observe them by walking around. The natural approach to a piece of art is visual perception [1]: looking at it and examining the details with our eyes, feeling emotions aroused by that view. However, a work of art is more than this. Paul Klee said that “the work of art is, above all, a process of creation”, and this process is enclosed mostly by the “surface” of the artwork. To clarify, we mean a physical surface and a physical texture.

An ancient oil painting, for example, is a thin stratigraphy (hundreds of microns to millimeters) made of heterogeneous materials that have been layered on a canvas or on a wooden support, typically including ground preparation, paint layers with pigments in an organic binder, and varnished upper layers [2]. The structure of the surface, in this case, is the result of the whole contributions, from the smooth deformation of the support to the asperities of the painting layer, to incisions and brushstrokes [3]. Moreover, the surface is also the most vulnerable part of the artwork because it is in contact with the external environment, making a piece of art a continuously changing system. The surface microstructure thus contains information about the decay due to microclimate interactions, such as crack patterns and detachments, or the modifications induced by restoration intervention, such as traces of cleaning treatments [4,5,6].

As mentioned, sight is the first sense involved in artwork fruition and, clearly, an ancient painting was conceived by the artist as visual artwork, but it is known that there is another sensor modality that can be meaningful: the touch [7]. We do not enter the intriguing debate about seeing with touch [8], as it is not the aim of this paper.

The focus of this research is on the importance of the information contained in the artwork surface, beyond the shape, and how to make this information accessible in a reliable way. In the framework above, we are motivated by the following observations:

- The artwork surface holds a lot of geometric information, being a superimposition of a large number of spatial wavelengths and having an intrinsic multiscale nature, from shape to local texture;
- Typically, the texture of ancient artworks, like 2D paintings and 3D archeological artifacts, exhibits distinctive features at submillimeter scales [9,10];
- As detailed later, human touch is sensitive to patterns and structures on scales of lengths of hundreds of micron [11,12];
- The emerging role of 3D survey and 3D printing in heritage applications [13].

Thus, our idea is to employ optical scanning profilometry, based on custom instrumentation optimized for artworks acquisition, with the aim of obtaining a high-quality dataset of the artwork surface at submillimetre scales (down to tens of microns). From this microsurface dataset, we will then extract the information suitable for tactile fruition through 3D printing technology.

Human tactile perception is sensitive in perceiving different materials. However, the interaction of the finger with the surface is a complex process that depends on the topography of the surface, frictional forces, and movement. Even if there is not a univocal threshold in the definition of the minimum feature size that can be detected, it seems that for the static touch, the limit is around 100–200 μm [11,12], whereas below 100 μm, the perception of the roughness is seriously degraded without movement. For the dynamic touch, the accepted threshold for the detection of the feature is in the micron range [12]. Sahli et al. [14] highlight that the tactile perception of similarity between surfaces is governed by the statistical microscale roughness rather than by their topographic resemblance, in contrast with the visual perception, which is dominated by the surface height topographic resemblance.

Artworks digitization has found a rapid expansion in museums, where it represents a useful tool not only for the insiders, such as restorers and conservators but also for a wider audience as, for example, visually impaired people that can reconstruct the mental image of the work through haptic fruition. The possibility of creating touchable reproductions for blind people was explored from the early nineties favoring the replica of 3D objects, while the challenging creation of representation of 2D artworks such as paintings was mostly realized starting from high resolution images [15,16,17,18]. Such tactile reproductions of 2D images introduce a bias in the interpretation of the original artwork [19]. Three-dimensional survey techniques allow overcoming this bias in the interpretation, although each method and technology has a resolution threshold and an effective performance that limits the smaller scale that can be “seen”.

Regarding the 3D acquisition of paintings, literature is found for the mostly used techniques based on commercial devices, from photogrammetry [20,21] to structured light and laser scanners [3,22], including Kinect sensors [23], also compared to microscopy [24], to the more recent reflectance transformation imaging technique [25].

In this work, we will perform 3D optical profilometry using a prototype, detailed later, that exploits conoscopic holography sensors to enable a versatile surface measurement with micrometric accuracy and micrometric resolution (depth and lateral) [26,27,28]. Laser scanning microprofilometry was demonstrated as effective for surface analysis in artworks (see the pioneering works [9,10]), including treatment monitoring [5].

A recent exemplar case study of 3D digitization, printing and fruition, using structured light scanner data, was proposed by Callieri et al. [29] on the painting Alchemy by Jackson Pollock, where the isolation of the geometrical component was done by exploring the model with the curator to emphasize the artist creation technique [30]. Three-dimensional printing technologies are gaining attention also thanks to the possibility to reproducing pieces of art for conservation reasons (see the recent review [13,31]).

Closing the above discussion, the prime concept is that if surface data are properly acquired, the information can be processed to tailor the different end-users applications, and 3D printing is the optimal tool that allows touching the information itself.

The main objectives of this work are summarized as follows:

- To demonstrate a workflow for tactile fruition of multiscale and polychrome artworks based on the acquisition of microgeometry and 3D printing technology;
- To validate the use of conoscopic holography sensors for high-quality acquisition of the surface dataset, up to the microscale, suitable for 3D printing.
- To demonstrate the potential of the tool not only for tactile fruition of artworks but also as an innovative resource for scientific “visualization” in conservation science.

It will be shown that scanning profilometry using conoscopic sensors allows capturing data with no geometrical distortion that are suitable for feature enhancement and optimization of the 3D printed texture for tactile fruition. A particular strength of the proposed method will be the capability of capturing color information in addition to the microscale surface features.

## 2. Materials and Methods

### 2.1. Optical Scanning Microprofilometry

Surface microprofilometry is performed with a custom device based on conoscopic holography and scanning techniques. The prototype was specifically designed to tailor the needs of cultural heritage applications [26], where target objects (from 2D paintings to 3D artifacts) have irregular shapes, deformations, and microtextures, with heterogeneous and polychrome materials, and the measurements (absolutely noninvasive) are performed in situ.

The conoscopic holography sensors, developed since 1980, are based on the analysis of an interference pattern [32,33]. Basically, the backscattered ray that comes from the target surface impinges on a uniaxial birefringent crystal causing splitting in the ordinary and extraordinary rays. In a first approximation, these two rays share the same geometric path but have different propagation velocities because the index of refraction for the extraordinary ray depends on the incident angle of the ray on the crystal. Once the rays exit the crystal, they interfere on the detector plane generating an interference pattern from which the distance measurement, at a micrometer scale, can be inferred. The main advantage of the interferometric sensors based on the conoscopic holography principle is their high stability, which makes them very suitable for measurements in out-of-lab environments, such as inside a museum.

The prototype developed in our laboratory integrates single-point probes with precision positioning stages for raster scanning the object and acquiring a surface map (heights data). This kind of distance measurement has not been widely investigated and has not yet been included in the classification surface texture measurement methods (ISO 25178-6 2010). The conoscopic holography probe setting [34] allows performing accurate measurements in different working ranges, which is a necessary condition in artworks applications [35], as shown below.

**Figure 1:**
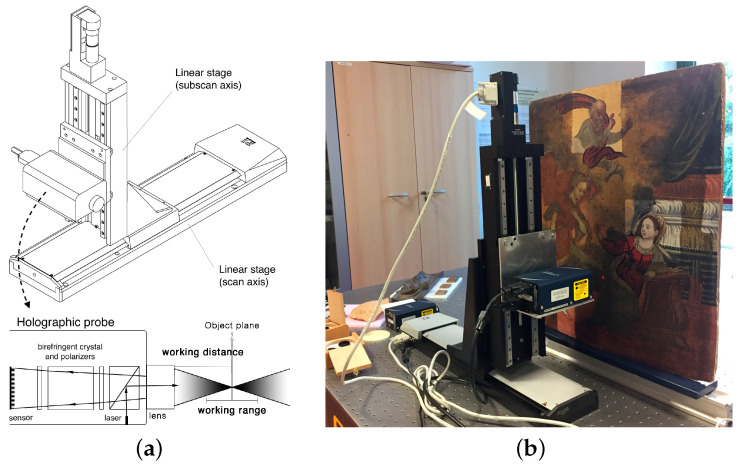
Microprofilometer vertical setup. (**a**) Schematic drawings with a scheme of the working principle of the probe. The orthogonal axes move the probe to scan the object plane at a safe stand-off distance; the depth measurement is effectively performed within the working range (or measurement range) of the sensor. (**b**) Application example in a real case study.

**Figure 2:**
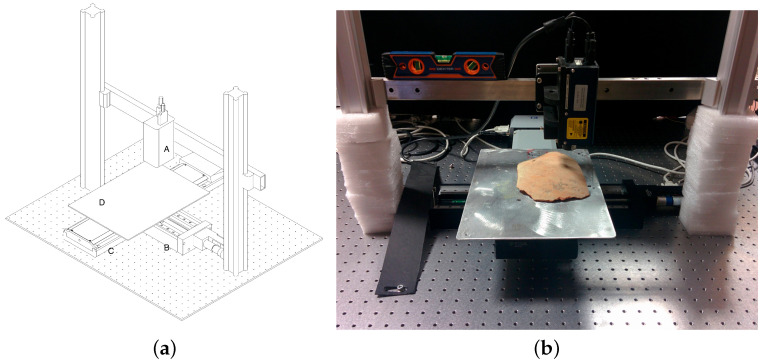
Microprofilometer horizontal setup. (**a**) sketch with the components specified. Conoscopic holography probe (A), linear stage (subscan axis) (B), linear stage (scan axis) (C), sample positioning plate (D). In this configuration, the probe is fixed while the object is moved by the axis stages in the horizontal plane. (**b**) application example in a real case study.

The linear stages (models M-4.14 and M-531 by PI) are orthogonally mounted to form the X-Y acquisition grid, allowing a maximum travel range of 300 mm and a step precision of 0.1 μm with an accuracy of 1 μm over the entire length. The X horizontal axis (main scanning direction) has a maximum scanning velocity of 50 mm/s while it is up to 3 mm/s for the sub-scan Y axis. The measurement session is performed by triggering the acquisition with a spatial domain pulsing technique, i.e., the sensor is triggered to the absolute position recorded by the axis. The use of scanning stages and a single-point collinear sensor, i.e., with no triangulation angle, allows for the effective measurement of deep concavities (up to a depth ten times the diameter) such as holes and grooves, e.g., the subtle incisions and craquelure found in ancient paintings. The stages can be assembled to perform the scanning in two different ways: the first configuration (Figure 1), in which the probe scans a fixed target, is suitable for in-situ analyses of standing large objects, while the second configuration (Figure 2), with the probe in a fixed position, is used for laboratory measurements of samples on the optical bench.

The characteristics that make the technique advantageous for cultural heritage applications are the portability of the prototype, which enables in situ diagnostics, and the versatility of the modular assembling, which allows for mounting different configurations of scanning stages and optical heads. This allows performing surface profilometry with high accuracy and high resolution on a wide (i.e., macroscopic) area ( 900 cm^2^), which is the key aspect when working on artworks. Moreover, the different lens-probe coupling enables different measuring ranges and hence the acquisition of objects of different shapes and deformation. As can be seen in Figure 1a, the working range is the maximum difference in surface height that does not affect the measurement. The prototype has been calibrated and optimized for working with several probes and lenses that allow acquiring different surfaces from micrometer texture details (e.g., working range of 0.6 mm with the ConoPoint-3HD and 25 mm lens) to 3D geometry (e.g., working range of 125 mm with the ConoPoint-3 and 200 mm lens).

In this work, we refer to accuracy as the depth (Z) accuracy of the sensor, defined as the difference between two flat surfaces measured as compared to the nominal value, while we refer to resolution as the sampling step (XY) of the scanning. The quality of each of the following measurements is assessed using the signal-to-noise ratio (SNR), the total signal collected (Total), and the correct working distance according to the lens–probe coupling [36].

### 2.2. Proof of Concept Case Studies

The following set of experiments was performed with the aim of investigating the feasibility of the laser scanning microprofilometry to acquire high-quality surface data on different kinds of artwork (paintings, relief, archaeological artifact) and then developing a method that allows the “translation” of the information of interest in innovative ways.

(1) Scientific fruition of paintings
  (a) Microsurface exploration for conservation scientists through 3D printing technology.
    Case study: oil painting on canvas by Tintoretto “St. Martial in Glory with the Saints Peter and Paul” in Venice;
    The specific aim is to optimize the signals without distorting the surfaces, turning them into “real objects” suitable to touch, thus providing a novel fruition tool for insiders.
  (b) Meaningful acquisition of a polychrome ancient painting in order to provide different ways of visualization and exploration of the microsurface information.
    Case study: eighteenth-century painting from private collection.
    The importance of this case lies in the information captured by the microprofilometer: both the heights map and the reflectance values are explored.
(2) In situ high-quality data acquisition in order to obtain accurate 3D printed replicas.
  Case study: “Dead Christ” bronze relief of the Donatello’s High Altar in the “Basilica del Santo” in Padua;
  This relief is an exemplar case study because it has multiscale surface features including main shapes (centimeters) with finer details (submillimeter) and the bronze texture (micrometers).
(3) Acquisition of painted figures on a 3D shape in order to process an enhanced replica that enables tactile exploration for visually impaired people.
  Case study: Apulian red-figure pelike (375–350 BC) exhibited at the Museum of Archaeological Sciences and Art in Padua;
  The technical importance of the case study is concerned with the acquisition of the macroscopic 3D shape and the microscale texture that encodes the figures represented on the vase, and their processing bearing in mind the haptic fruition.

Table 1 specifies the probe-lens coupling for each case study with the corresponding stand-off distance, working range, accuracy, and laser spot (nominal values). The trade-off on the precision (namely, on depth accuracy and laser spot) is determined by the choice of the working range, the most important factor when scanning 3D shapes or large paintings that have deformations and thick, non-homogeneous painting layers. Higher performance configuration can be used, at the cost of a shorter measuring range, in laboratory acquisition of flat paintings.

## 3. Results and Discussion

### 3.1. Scientific Fruition of Paintings

Conservation scientists work with the microsurface of the artworks, operating in direct contact with it. Thus, the natural question that arises is how the surface changes when a treatment is completed. A critical example is the cleaning process, an important step in restoration aimed at removing the degradation products in the upper surface layer [5]. However, the meaning of the microscale physical descriptors is not always easy to grasp. In the context of “seeing with touch”, the surface can be a great aid for conservators and scientists.

#### 3.1.1. Microsurface Exploration for Conservation Science: St. Martial in Glory with the Saints Peter and Paul by Tintoretto

Here we focus our attention on a laser and chemical cleaning treatment carried out on the oil painting on canvas by Tintoretto “Martial in Glory with the Saints Peter and Paul” in Venice. The aim of the treatment was to guarantee a controlled and selective removal of the varnish layers using a Er:YAG laser in a region of azurite mixed with calcium carbonate, lead white, red ochre, and carbon black [37].

**Figure 3:**
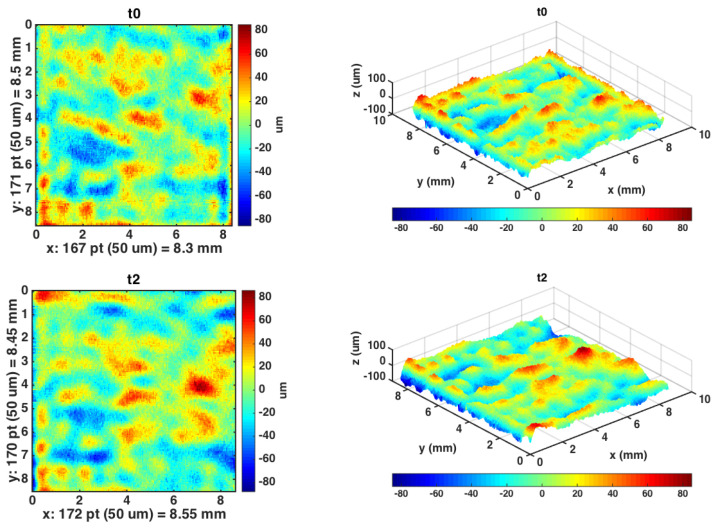
Surface data plotted as 2D height map of T0 and T2 and its 3D visualization.

The microprofilometer was used to acquire an ROI (Region Of Interest) of around 1 cm^2^ in a temporal sequence: before the treatment (T0), and after the final chemical treatment (T2). As can be seen in the resulting surface maps (Figure 3), the instrument was very powerful in acquiring the evolution of the microsurface. The microprofilometer was set in the vertical configuration (Figure 1) with the lens-probe coupling specified in Table 1. The sampling step was 50 μm and the scan velocity was set at 5 mm/s.

From a visual inspection of the micrometric height maps in Figure 3, small changes of the surface can be noticed. Looking at surface data from a statistical point of view, we estimated the overall roughness as the root mean square deviation ($S_{q}$) of the heights distribution in the entire ROI, after removing the large-scale tilting, and found similar values ($S_{q}∼\;$ 2.0 × 10^1^ μm) for the original surface and after the final chemical cleaning.

Now the question is: do we have other ways to describe and translate the information coming from the surface? In order to solve this issue, we elaborate on the surface data for optimizing the signals for the 3D printing.

**Figure 4:**
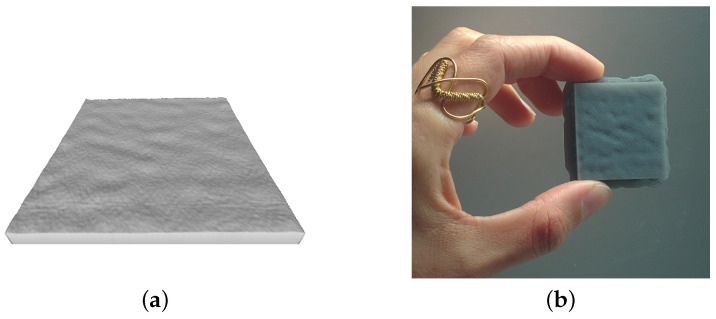
(**a**) Mesh of the enhanced surface T0 obtained from direct tessellation of the point cloud. This image represents the 3D printable STL file of the increased surface generated from the data. (**b**) 3D printed usable replica.

Figure 4a shows an example of the enhanced 3D simulation of the surface T0. All the meshes were then 3D printed using the Stereolithography (SLA) technology, choosing the maximum possible z-resolution of 50 μm in the printer settings, i.e., the minimum layer thickness in the z-axis direction: in Figure 4b there is an example of the result obtained using a photopolymeric resin.

**Figure 5:**
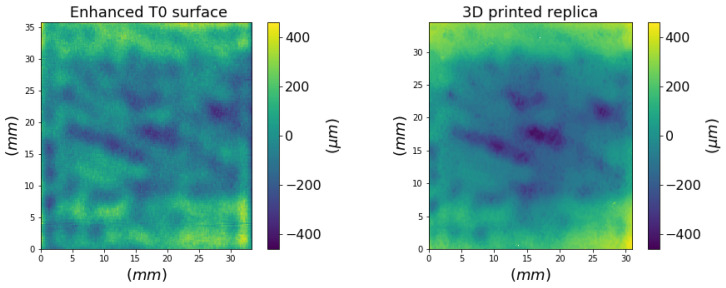
Surface height map of the enhanced surface T0 and its 3D printed replica.

Taking the original surface (T0) as a reference example, Figure 5 depicts the comparison between the surface maps acquired on the replica with the profilometer and the original data (the enhanced ROI) used for the 3D printing. An isotropic enhancement was studied in order to obtain an overall roughness value suitable for the tactile exploration (greater than 100 μm) and reproducible by the 3D printer. We obtained an $S_{q}$ of 1.2 × 10^2^ μm for the enhanced original surface and of 1.5 × 10^2^ μm for the 3D replica.

**Figure 6:**
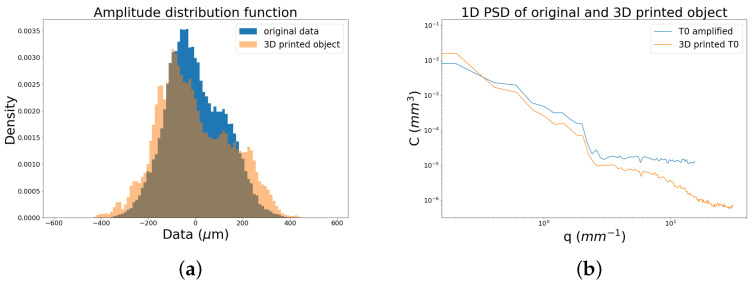
Signal of the enhanced surface and 3D printed replica: (**a**) height distribution and (**b**) power spectrum.

The 3D printing process adds a step in the chain that starts from the original surface, passes through the acquisition, and concludes with the 3D printing with the consequent modulation of the signal. Figure 6 highlights the differences between the original signal and its replica in terms of the Amplitude Distribution Function (ADF) and in terms of spatial frequencies ($q_{i}=2\pi /\lambda_{i}$) as Power Spectral Density (PSD). The 3D replica was scanned with a double frequency in order to allow a meaningful comparison with the original data. As can be seen, the ADF of surface data of the replica has a greater width showing a higher density in the large amplitudes compared to the original surface signal. It is noticeable in the PSD the loss of signal in the high frequencies of the 3D printed object. We observe that the trend of the PSD is preserved up to a scale of λ∼ 630 μm corresponding to a spatial frequency of q∼10 ${\mathrm{mm}}^{-1}$, showing a preservation of the multiscale features of interest, while there is a degradation of the finer roughness. However, such surface asperities are not considered representative of any texture pattern induced by the treatment in the painting (non-amplified) surface.

The experiment discussed is a proof of concept. We may conclude that the microprofilometry coupled to 3D printing technology can be an innovative tool for the scientific visualization in conservation. Thanks to an isotropic rescaling of the surface that conserves the statistical properties, the enhanced replica allows a visual (naked eye) and a haptic approach to the fruition of the surface texture data at a microscale, which is informative for restoration treatment monitoring.

#### 3.1.2. High Precision Microgeometries Acquisition and Exploration: Polychrome Eighteenth-Century Painting

The other part of the experiment involves an eighteenth-century painting belonging to a private collection. This painting was subjected to some experimental laser cleaning tests that have lowered the surface by removing the upper layer.

In this case, it was possible to carry out laboratory measurements on an optical bench, as shown in Figure 1. The acquisition was performed on a cleaned region of the painting using the high precision conoscopic holography probe (HD) specified in Table 1. The scanning sampling step was set at 50 μm and the scan velocity at 10 mm/s. Some details of the painting were also sampled with a finer scan step of 25 μm.

**Figure 7:**
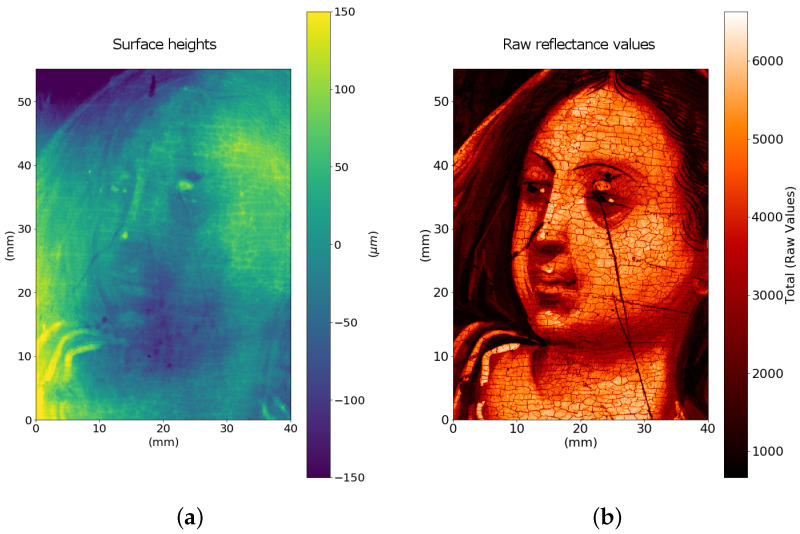
(**a**) Surface height dataset and (**b**) raw reflectance total dataset of an ROI of the painting acquired by the microprofilometer at a 50 μm sampling step. The two maps are spatially registered at micrometric precision.

The acquired data are very meaningful, both the surface heights and the raw reflectance total dataset provided by the conoscopic measurement, with the latter containing the laser intensity values just after the backscattering of the beam from the surface (see Figure 7). The microstructure of the brushstrokes is enclosed within about 300 μm, a range suitable to tactile sensitivity, and the raw reflectance values add another useful scientific piece of information: from Figure 7b, it can be seen that, in addition to some color information, the significant craquelure of the painting is highlighted, allowing a scientific visualization of the fine meaningful details. On this regard, it is worth noting that, as the wavelength of the laser beam is 655 nm, the red pigments have high reflectance while the craquelure pattern is effectively detected because the reflectance drops to zero. The strength of a joint exploration of these two datasets, which are spatially registered at micrometric precision, is meaningful: on the one hand, the reflectance map allows a visualization of the craquelure, and on the other hand, the surface heights map enables micrometric measurements of the craquelure itself.

**Figure 8:**
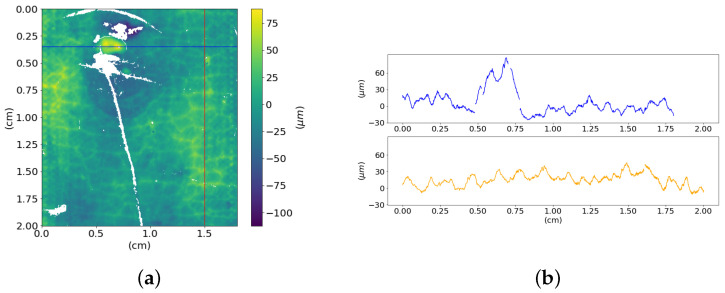

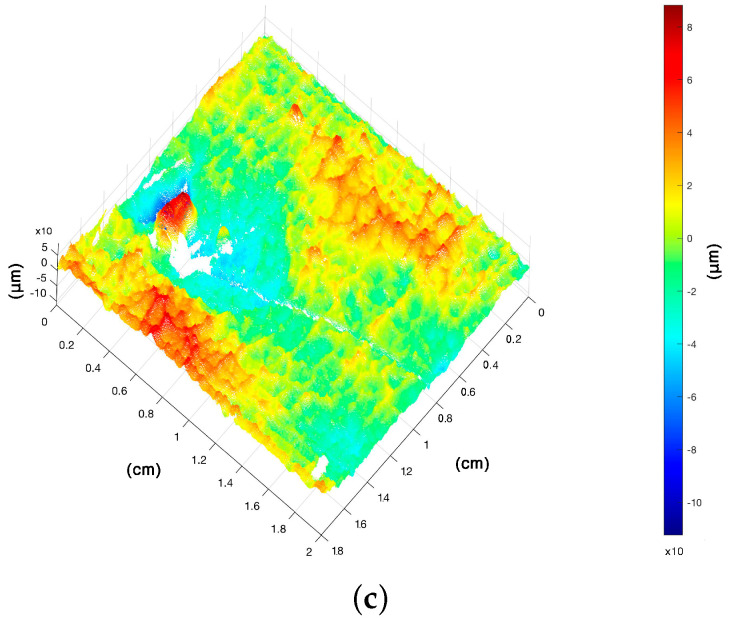
Surface data of an ROI around the eye sampled at 25μm step: (**a**) 2D height map, (**b**) line profiles of the highlighted row (blue) and column (orange) and (**c**) representation of data as a point cloud in space that can be interactively explored by the insiders.

Figure 8 depicts a useful way for the conservation scientist to visualize and to explore interactively the micrometric information provided by the profilometer, by displaying the surface through the point cloud. In particular, we selected an ROI of about 4 cm^2^ around the eye of the figure, sampled at 25 μm: the craquelure decay pattern of the painted layer is evident. Surface asperities are in the scales of tens of micrometers, as can be seen in Figure 8b, where two profiles are plotted. The overall root mean square $S_{q}$ is ∼ 20 μm.

In summary, optical profilometry captures meaningful information also on a polychrome painting. In order to obtain a replica of the surface texture suitable for tactile exploration, taking into account the limited z-resolution of the 3D printing technology, an enhancement of the dataset with a magnification factor higher than 7× is necessary.

### 3.2. High-Quality 3D Printed Replica: Donatello’s Dead Christ Bronze Relief

**Figure 9:**
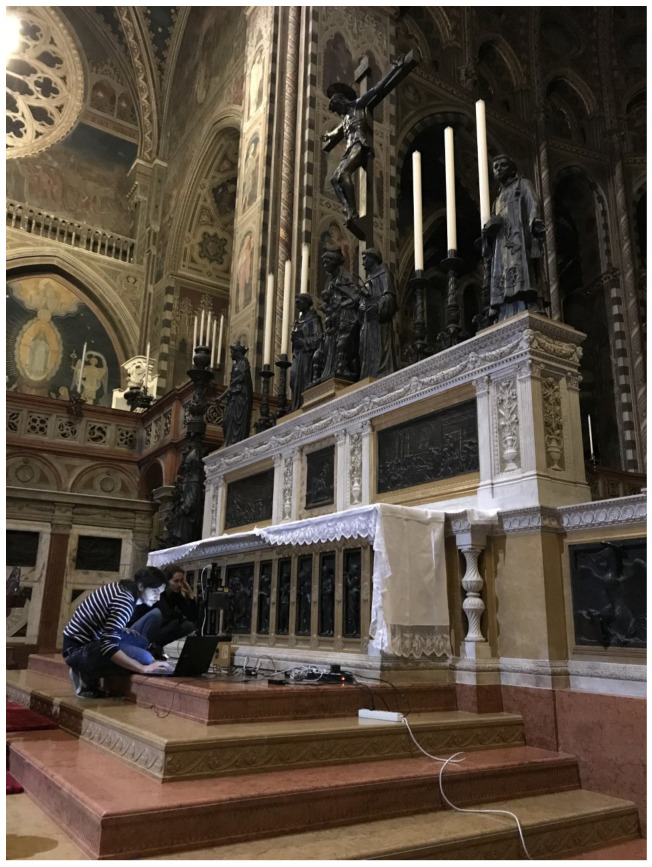
Measurements performed in situ at the Donatello’s High Altar in the “Basilica del Santo” in Padua.

The method was tested on the notable *Dead Christ* bronze relief (1453) of the Donatello’s High Altar in the “Basilica del Santo” in Padua, as shown in Figure 9.

The microprofilometer was configured for in situ measurements in a vertical setup with the probe-lens coupling reported in Table 1. Raster scanning was performed with a sampling step of 100 μm and a scan velocity of 10 mm/s. As the Santo is a tourist place, the measurements were carried out during the evening to avoid vibrations caused by the visitors.

**Figure 10:**
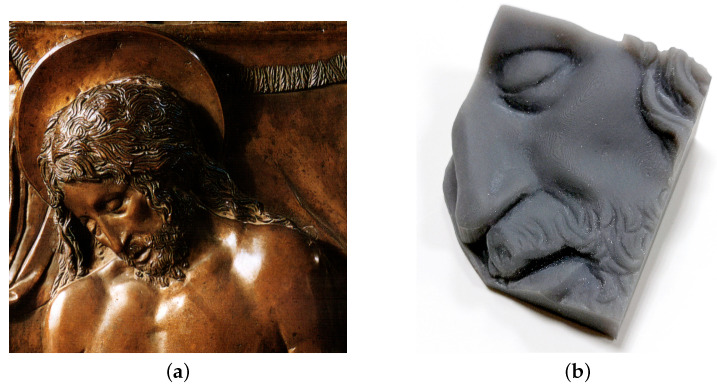
(**a**) Detail of the *Dead Christ* bronze relief by Donatello. (**b**) 3D printed replica of the acquired ROI of the face.

Optical scanning microprofilometry was very effective in acquiring the bronze relief, providing information both on the shape of the sculpted elements than on the surface texture. Here, surface data were used to create a 3D printed replica of the object as it is, i.e., without any dimension scaling or information enhancing. As seen in Figure 10, the final product is very satisfactory and the finest details are well reproduced.

**Figure 11:**
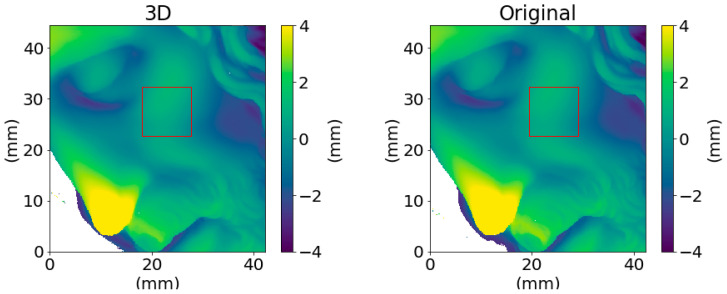
Height map of the 3D printed replica and original object acquired by the microprofilometer. The red areas highlight the smaller ROIs used to compare the texture parameters.

In order to validate the method, the 3D printed object was acquired with the microprofilometer and the two surface datasets (original and replica) compared. Figure 11 reports the surface data as 2D height maps. Examining a smooth ROI of ∼ 1 cm^2^ around the cheekbone of the *Dead Christ* and its 3D replica, highlighted in red in Figure 11, it can be observed that the amplitude texture parameters are comparable. The root mean square deviation $S_{q}$ is ∼8 × 10^1^ μm for the original object and ∼7 × 10^1^ μm for the 3D replica, while, computing the high-order statistics of the ROIs, we have a skewness of 0.21 for the original data and of 0.22 for the replica and kurtosis values of 5.5 and 4.3, respectively.

**Figure 12:**
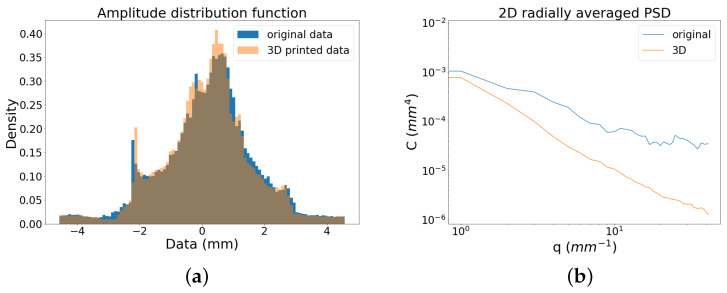
Signal of the original surface and 3D printed replica: (**a**) height distribution and (**b**) power spectrum.

In Figure 12b, the broadband surface signals of the original object and the replica (entire dataset) are compared in terms of their frequency content using the power spectrum. The PSD allows an analysis of the in-band roughness, i.e., in relation to the multiscale features of the bronze relief. It is evident the drop in the PSD of the 3D printed object not only in the higher frequencies attributable to the surface texture but also in the mid components attributable to the hair and the beard shapes. In this context, it is worth noting that the printing process occurs in the following steps: the replica grew upside down during the printing and then it underwent washing and post-curing processes. During these steps, it is possible that some liquid drops were trapped within the valleys smoothing the surface. This is also in agreement with the significant decrease observed in the peak to valley distance of the printed replica as well as in the variation in the kurtosis, i.e., with a general smoothing of the surface.

Despite the slight signal degradation introduced by the 3D printing process, we conclude that the high accuracy and high resolution of the data acquired with the profilometer have allowed obtaining a significant result, in which the information regarding the most representative multiscale features of the artwork is preserved.

**Figure 13:**
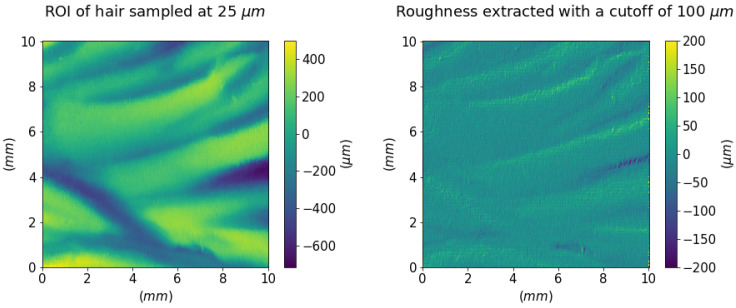
Height map of a portion of Christ’s hair sampled at 25 μm and its finer surface texture.

Figure 13 (left) shows a meaningful ROI of 1 cm^2^ of the hair of the *Dead Christ* relief sampled at a 25 μm step. This higher resolution acquisition allows appreciating the details of the incisions that are the essence of the artwork itself. The grooves lie in a range of about 50–600 μm, which is enough for the sensitive range of human tactile perception [11]. Figure 13 (right) shows the higher frequencies separated from the shape using a Gaussian filter with a cut-off of 100 μm. This signal separation enables estimating the texture $S_{q}∼$2 × 10^1^ μm. As expected, the bronze roughness signal is too low for our touch, and we perceive just a smooth (i.e., with no texture) surface.

### 3.3. Tactile Exploration of Painted Figures for Visually Impaired People: Apulian Red-Figure Pelike

**Figure 14:**
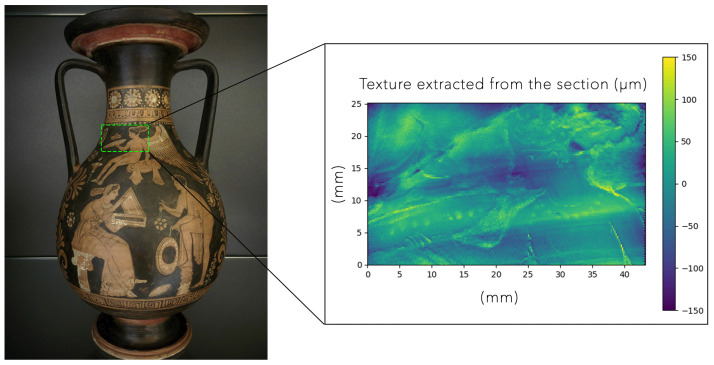
Red-figure pelike with the investigated ROI highlighted. The zoom shows the texture surface data, plotted as a height map, once the shape of the vase is removed.

The third experiment is in regard to an Apulian red-figure pelike attributable to the artistic production of the Terrytown Group (375-350 BC) exhibited at the Museum of Archaeological Sciences and Art in Padua (Figure 14). Thus, the challenge and the purpose of this case are to make the vase and the painted figures readable for visually impaired people.

As in the previous cases, the microprofilometer was configured for in situ measurements, with the specifications of Table 1. The working range and the accuracy allowed to scan a significant ROI of the vase, measuring from the macroscopic 3D shape up to the fine details of the texture. The scanning sampling step was set to 200 μm, and the scan velocity to 10 mm/s. Some details of the vase were also sampled with a finer scan step of 50 μm.

Figure 14 shows the surface texture information with the data displayed as a height map. The texture signal was obtained after form removal with a polynomial fitting, resulting in a “flat” surface with an overall $S_{q}$∼ 41 μm. The height difference in the transition from the red to the black color was estimated to be ∼ 40 μm. Computing the amplitude parameters, we found that the maximum height values ($S_{z}$) of the red and the black parts are different, ∼1.9 × 10^1^ and ∼1.3 × 10^2^ μm, respectively, while the maximum valley depth values ($S_{v}$) are comparable and in the order of ∼2.6 × 10^1^ and ∼1.3 × 10^1^ μm, respectively. The result was confirmed by the archaeologist, reporting that the Red-figure technique implies that the figures were first carved and sketched in clay and then the external parts were covered by a black engobe.

**Figure 15:**
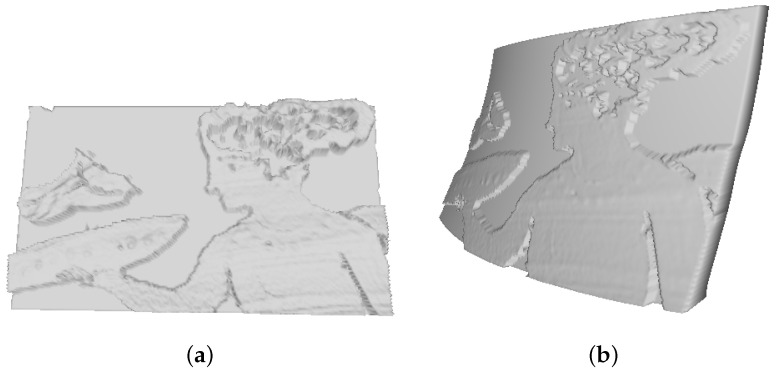
(**a**) Printing simulation of the red figure with an amplified texture, placed in relief on a plane once it is extracted thanks to the reflectance values. (**b**) Printing simulation of the extracted red figure placed in relief on the original shape.

As shown by Gaburro et al. [26], by exploiting the raw total signal recorded by the detector, the different colored regions can be discriminated, point-wise in the scan step sampling grid. This way, the texture information can be accurately selected and the features of interest enhanced. Figure 15 shows the meshes developed for the haptic fruition with the red figure raised by 1 mm from the main shape, thus making the information easily accessible for the tactile exploration. The selected amplified texture can be applied on a plane or to the original shape and 3D printed.

We conclude that the scanning profilometry method is effective in acquiring texture information with extended morphological features and surface heights on scales of tens of micrometers, such as the figures decorating the vase.

## 4. Conclusions

In this study, we have proposed and validated a method for scientific exploration and haptic fruition of the microsurface of artworks based on optical scanning profilometry. It is demonstrated that the combination of accurate micrometric data, acquired without distortion, and 3D printing is a powerful tool that allows not only a tactile fruition of the artwork but also an innovative way to scientifically visualize the surface in the field of conservation science.

The human touch is sensitive to the scale of ∼ 100 μm and an important role in the tactile perception is covered by the statistical microscale roughness. Following this assumption, we collected meaningful data, namely the microscopic textures of exemplary artworks together with their macroscopic shape, concentrating also on the analysis of the statistical descriptors related to the average behavior of the surface heights. The purpose was the “translation” of the information of interest in new usable ways suitable to the touch, exploiting available 3D printing technology. In particular, working on real ancient artworks, we carried out three different experiments that have led to new fruition ideas. The tests are meant as a proof of concept, demonstrating the feasibility of the method in meaningful ROIs of the artworks, taking into account the limit of the working range of our instrument.

The first experiment regarded new approaches to access the information of the paintings. On one side, it demonstrated that surface data can be isotropically enhanced and 3D printed while conserving the statistical properties and becoming touchable for conservation scientists. On the other side, it introduced new ways to explore the microgeometry of a polychrome painting. The second test validated the use of the optical profilometer to obtain a detailed 3D printed replica of an artwork, in the specific case, a relief. In the third experiment, we reached the goal of making a thin painted figure suitable for the haptic fruition while conserving the texture information and the shape of the original object, in the specific case, an ancient vase.

Depending on the objective to be achieved, one can decide if the surface needs to be enhanced or not. For example, the bronze surface of Donatello’s relief is visually perceived as smooth and indeed the roughness is very low. In this case study, the importance lies in the very detailed acquisition and reproduction of the 3D sculpted shapes down to the submillimeter scale. Starting from a surface dataset acquired with a sampling step of 50 μm and depth accuracy of 10 μm, in a measurement range of centimeters, an accurate 3D printed replica was obtained using available SLA technology. Differently, the texture of the two ancient paintings is visually perceivable, with the brushstrokes on the ∼ 100 μm scale and a finer root mean square roughness $S_{q}∼$ 20 μm. This implies that the translation of this data into a tactile experience, highlighting the information trapped within the surface both for insiders and for visually impaired people, needs the enhancement of the surface texture, consistently with the 3D printing resolution, in order to reach the scale of touch sensitivity. This is true also for the painted figures of the Greek vase where the roughness is ∼ 40 μm. Here, the surface heights of the colored areas have been raised up to the millimeter from the curved original shape.

Overall, the powerful starting point of each application was the high resolution and high accuracy of the data acquired by the optical scanning microprofilometer based on conoscopic holography, which allows capturing the texture of the surface, the fundamental signal for “seeing” with touch.

## Figures and Tables

**Table 1:** Summary table of the sensor parameters for each case study.

Case Study		Probe	Lens	Stand-Off Distance	Working Range	Accuracy	Laser Spot
				(mm)	(mm)	(μm)	(μm)
1 (a)	Painting	ConoPoint-3	100 mm	95	35	15	63
	(in situ)					
1 (b)	Painting	ConoPoint-3HD	50 mm	42	2	2.5	19
	(lab)						
2, 3	3D shape	ConoPoint-3	75 mm	70	18	10	47
	(in situ)						

## Data Availability

The data acquired on the artworks are not available for free access.

## Abbreviations

The following abbreviations are used in this manuscript:

SNR	Signal-to-Noise Ratio
ROI	Region Of Interest
ADF	Amplitude Distribution Function
PSD	Power Spectral Density
SLA	Stereolithography Apparatus
